# Changes in plasma levels of perfluoroalkyl substances (PFASs) are related to increase in carotid intima-media thickness over 10 years – a longitudinal study

**DOI:** 10.1186/s12940-018-0403-0

**Published:** 2018-07-03

**Authors:** P. Monica Lind, Samira Salihovic, Jordan Stubleski, Anna Kärrman, Lars Lind

**Affiliations:** 1 Department of Medical Sciences, Occupational and Environmental Medicine, Uppsala University, 751 85 Uppsala, Sweden; 2 Department of Medical Sciences, Molecular Epidemiology and Science for Life Laboratory, Uppsala University, Uppsala, Sweden; 3 MTM Research Center, School of Science and Technology, Örebro University, Örebro, Sweden; 4 Department of Medical Sciences, Cardiovascular Epidemiology, Uppsala University, Uppsala, Sweden

**Keywords:** Atherosclerosis, Longitudinal, Perfluoroalkyl substances (PFASs), Elderly, Epidemiology, IMT

## Abstract

**Background:**

It has previously been reported that the environmental contaminants perfluoroalkyl substances (PFASs) are linked to atherosclerosis in cross-sectional studies. Since cross-sectional studies could be subject to reverse causation, the purpose of this study was to analyze if the longitudinal changes in PFASs during a 10-year follow-up were related to the change in carotid artery intima-media thickness (IMT, ultrasound) during the same period.

**Methods:**

In the Prospective Investigation of the Vasculature in Uppsala Seniors (PIVUS) study, 1016 individuals were investigated at age 70; 826 of them were reinvestigated at age 75 and 602 at age 80 years. Eight different PFASs were measured in plasma by ultra-performance liquid chromatography-tandem mass spectrometry (HPLC-MS/MS), and IMT was measured at all three time points. Random-effects mixed regression models were used to examine the associations over time.

**Results:**

IMT increased 0.058 mm during the 10-year period (*p* < 0.0001). Following adjustment for baseline values of PFASs (age 70) and sex, the changes in plasma levels of 6 of the 8 measured PFASs were significantly related to the change in IMT over the 10-year follow-up period in a positive fashion (*p* < 0.0062 using Bonferroni correction for 8 tests). Further adjustment for traditional cardiovascular (CV) risk factors (HDL and LDL cholesterol, smoking, systolic blood pressure, statin use, fasting glucose and serum triglycerides) affected these relationships only marginally.

**Conclusion:**

The change in plasma levels of several PFASs during 10 years was positively related to increase in IMT seen during the same period, giving prospective evidence that PFASs might interfere with the atherosclerotic process.

## Background

Perfluoroalkyl substances (PFASs) belong to a class of organic environmental contaminants that are structurally characterized by a perfluorinated carbon backbone with a terminal functional head group. They are produced in high volumes and used in a variety of applications, ranging from surface-active repellents to fire-fighting foams and cosmetics. Due to their wide use, PFASs have been distributed globally and are detected in blood in almost all individuals in industrialized countries [1–10].

Atherosclerosis is a common disease affecting the majority of individuals in the industrialized countries and could give rise to myocardial infarction and stroke. Several imaging methods to quantify the amount of atherosclerosis are used in research and clinical practice. Of these, ultrasound determinations of the carotid arteries are very common in research; especially the thickness of the intima-media (IMT) complex of the carotid artery wall has been evaluated in many epidemiological studies, and increased IMT has been associated with future myocardial infarction and stroke [11].

One previous publication has reported an association between circulating levels of PFASs and IMT using a cross-sectional approach [12]. Since cross-sectional studies might be subject to reverse causation and confounding, longitudinal studies are preferable. We therefore now present data from the Prospective Investigation of the Vasculature in Uppsala Seniors (PIVUS) study [13], in which the participants were investigated at ages 70, 75 and 80 years. Eight different PFASs and ultrasound-based IMT were measured at the three time points, and the study investigated the hypothesis that PFASs levels were positively related to the change in IMT over the 10-year follow-up.

## Methods

### Subjects

Subjects eligible for inclusion were all individuals aged 70 years living in the municipality of Uppsala, Sweden. The subjects were selected from the municipal register and invited in a randomized order by letter within 2 months of their 70th birthday. Of the 2025 subjects invited, 1016 took part, yielding a participation rate of 50.1%. This baseline investigation was conducted between 2001 and 2004 [13]. All subjects were invited to re-examinations at ages 75 (*n* = 826) and 80 years (*n* = 602). During the 10-year follow-up, 153 had died.

The study was approved by the Ethics Committee of Uppsala University, and the participants gave informed consent prior to the study.

### Basic characteristics and cardiovascular risk factors

The participants were asked to complete a questionnaire concerning their medical history, smoking habits, and regular medication. All subjects were examined in the morning, having fasted overnight. No medication or smoking was permitted after midnight. Blood pressure was measured using a calibrated mercury sphygmomanometer in the non-cannulated arm to nearest mmHg after at least 30 min of rest, and the average of three recordings was used. Lipid variables and fasting blood glucose were determined using standard laboratory techniques [14]. The same methods were applied at all three examinations. Basic cardiovascular risk factors are presented in Table 1 and have been described more in detail in [13].

**Table 1:** Traditional cardiovascular risk factors at the three different investigations in the PIVUS sample

	Age 70	Age 75	Age 80
Variable	Mean (SD)	Mean (SD)	Mean (SD)
BMI (kg/m^2^)	27.0 (4.3)	26.8 (4.3)	26.9 (4.5)
Fasting glucose (mmol/l)	5.3 (1.6)	5.2 (1.4)	5.2 (1.3)
Systolic blood pressure (mmHg)	149 (22)	148 (19)	146 (19)
LDL cholesterol (mmol/l)	3.38 (0.88)	3.37 (0.94)	3.2 (0.9)
HDL cholesterol (mmol/l)	1.51 (0.43)	1.49 (0.46)	1.38 (0.39)
Smokers (%)	11	6	3
Statin use (%)	15	25	30

*BMI* body mass index, *SD* standard deviation, *HDL* high-density lipoprotein, *LDL* low-density lipoprotein

At baseline, approximately 10% of the cohort reported a history of coronary heart disease, 4% reported stroke and 9% diabetes mellitus. Almost half the cohort reported some cardiovascular medication (45%), and antihypertensive medication was the most prevalent (32%). Fifteen percent reported use of statins, while insulin and oral antiglycemic drugs were reported in 2 and 6%, respectively (for details, see reference [13]).

### Carotid artery ultrasound evaluation

The carotid artery was assessed by external B-mode ultrasound imaging (Acuson XP128 with a 10 MHz linear transducer, Acuson, Mountain View, CA). The images were digitized and imported into the AMS (Artery Measurement Software) automated software for dedicated analysis of IMT in the far wall in the common carotid artery (CCA) 1–2 cm proximal to the bulb. A 10-mm segment with good image quality was chosen for analysis. The program automatically discerns the borders of the intima-media thickness of the far wall and the inner diameter of the vessel and computes intima-media thickness and the diameter from roughly 100 discrete measurements through the 10-mm-long segment. The value given for IMT is the mean value from both sides. The measurements of intima-media thickness were repeated in 30 random subjects yielding a coefficient of variation of carotid artery intima-media thickness of 7.2% [15].

### Methods for the chemical analysis of the PFASs

The analytical procedure used for all plasma samples (150 μL) in the present study involves rapid protein and phospholipid removal using Ostro 96-well plates (Waters, Milford, MA). Following extraction, the samples were transferred to vials and analyzed using large volume injection (LVI) automated column-switching ultra-performance liquid chromatography-tandem mass spectrometry (HPLC-MS/MS, Waters, Milford, MA). In total, the method enabled the assessment of 14 PFASs. For this study, however, only PFASs with detection rates > 75% were used in subsequent statistical analyses: perfluoroheptanoic acid (PFHpA), perfluorononanoic acid (PFNA), perfluorodecanoic acid (PFDA), perfluoroundecanoic acid (PFUnDA), perfluorohexane sulfonic acid (PFHxS), linear isomer of perfluorooctane sulfonic acid (PFOS), perfluorooctane sulfonic acid (PFOA) and perfluorooctane sulfonamide (PFOSA). Detailed information about the analytical procedure and method performance is presented in [16].

### Statistical analyses

All eight PFASs were skewed towards high levels but were normally distributed following ln-transformation. The same trend was also observed for fasting blood glucose and serum triglycerides. Therefore, the ln-transformed values were used in the statistical models.

First, the change in IMT over 10 years (three measurements) were evaluated by mixed random effect models with IMT as dependent variable, and time as the independent variable and sex as confounder (age same in all subjects).

Thereafter, the relationships between changes over 10 years in the levels of the eight PFASs and the changes over 10 years in IMT were examined. Also for this purpose mixed random effect models were used, but here each PFAS was used as an independent variable. The independent variable was split into a between-individual component, which is the first observation for the individual, and a within-individual component, which is the difference between the measurements at future time points and the first measurement. Thus, the between-individual component is related to the mean of the three measurements of IMT, while the between-individual component, as a single term, relates the change in each PFAS to the change in IMT. The theory and assumptions behind this model as well as the detailed formula is given at page 420 in Applied longitudinal analysis by Fitzmaurice, G.M. et al. [17]. The general formula is; *Y*_*ij*_ = *Z*_*i*_*beta*_0_ – *X*_*i*1_*beta*_*C*_ + (*X*_*ij*_ − *X*_*i*1_)*beta*_*L*_  + *e*_*ij*_, where Y is IMT, X is the PFAS, i is the individual, j the time, beta_C_ is the coefficient for the first observation and beta_L_ is the coefficient for change over time. Confounders and the intercept are given as Z_i_beta_0._ The general formula we used in STATA for the first set of models was; mixed IMT PFASchange PFAS1 sex || id:, where 1 denotes the first observation at age 70. We used only random intercept, since inclusion of also a random slope made the models not to converge in most cases. The random part of the model is the identity of the subjects and the rest of the model are fixed effects. We used the default covariance structure of the command “mixed”, which is independent, which is appropriate for mixed models with a random intercept.

The first set of models (one for each PFAS) were adjusted for sex and the baseline values for the PFASs. The second set of models were also adjusted for traditional cardiovascular risk factors (HDL and LDL cholesterol, smoking, systolic blood pressure, BMI, fasting glucose and serum triglycerides) and statin use.

An interaction term between sex and the PFASs was included in the first set of models to determine any sex interaction regarding IMT.

STATA14 (Stata Inc., College Station, TX) were used for calculations. Since we investigated 8 PFASs, we applied *p* < 0.00625 (0.05/8) as the level of significance.

## Results

Distributions of traditional CV risk factors are summarized in Table 1. Median IMT at age 70 was 0.89 mm (SD 0.16). An increase in IMT by 0.058 mm (SD 0.043, *p* < 0.0001) was observed during the 10-year follow-up. This is well in line with a meta-analysis of 22 studies in which the annual change in IMT ranged from 0.001 to 0.030 mm [18].

The baseline levels of the PFAS are given in Table 2. The results of the longitudinal changes in the eight PFASs have been given in detail in a previous publication [19]. In summary, over the 10- year follow-up, PFHxS, PFUnDA, PFDA, and PFNA levels increased (ranging from 7 to 34%), while PFOA, PFHpA, PFOS, and PFOSA levels were reduced (ranging from − 75% to − 27%) in a significant fashion.

**Table 2:** Median and interquartile range for the eight studied PFAS at the baseline investigation (at age 70) in ng/ml

PFHpA	0.05 (0.03, 0.09)
PFHxS	2.08 (1.6, 3.42)
PFOS	13.23 (9.95, 17.77)
PFOA	3.3 (2.52, 4.39)
PFNA	0.70 (0.52, 0.97)
PFDA	0.31 (0.24, 0.40)
PFOSA	0.11 (0.07, 0.17)
PFUnDA	0.28 (0.22, 0.37)

PFASs: *PFHpA* perfluoroheptanoic acid, *PFHxS* perfluorohexane sulfonic acid, *PFOS* perfluorooctane sulfonic acid, *PFOA* perfluorooctane sulfonic acid, *PFNA* perfluorononanoic acid, *PFDA* perfluorodecanoic acid, *PFOSA* perfluorooctane sulfonamide, *PFUnDA* perfluoroundecanoic acid

A Spearman rank correlation matrix for the changes from age 70 to age 80 regarding the 8 PFAS are given in Table 3.

**Table 3:** Spearman rank correlation for relationships between the changes from age 70 to age 80 for the different PFAS studied

	PFHpA	PFHxS	PFOS	PFOA	PFNA	PFDA	PFOSA
PFHxS	0.15						
PFOS	0.20	0.20					
PFOA	0.35	0.35	0.50				
PFNA	0.19	0.22	0.35	0.46			
PFDA	0.18	0.20	0.38	0.41	0.75		
PFOSA	0.20	0.04	0.36	0.28	0.12	0.13	
PFUnDA	0.09	0.21	0.31	0.31	0.57	0.76	0.09

PFASs: *PFHpA*, perfluoroheptanoic acid, *PFHxS* perfluorohexane sulfonic acid, *PFOS* perfluorooctane sulfonic acid, *PFOA* perfluorooctane sulfonic acid, *PFNA* perfluorononanoic acid, *PFDA* perfluorodecanoic acid, *PFOSA* perfluorooctane sulfonamide, *PFUnDA* perfluoroundecanoic acid

The changes in plasma levels of all eight PFASs over the 10 years were related to the change in IMT during the same period (when analyzed one by one), adjusting for baseline levels of the PFASs and sex, when *p* < 0.05 was used. Using a Bonferroni-corrected *p*-value for eight tests (*p* < 0.00625), six of the eight PFASs were still significant, as can be seen in Table 4.

**Table 4:** Relationships between changes in plasma levels of eight PFAS over 10 years and change in IMT over 10 years. Both the PFASs and IMT were measured at age 70, 75, and 80 years. All models were adjusted for sex. In the multiple adjusted models, additional adjustment was performed for HDL and LDL cholesterol, smoking, systolic blood pressure, statin use, BMI, fasting glucose and serum triglycerides at the three time points

	Beta	SE	z	p-value	[95% Conf. Interval]	Change in IMT for an IQR change in the PFAS
PFHpA sex-adjusted	0.010	0.003	3.05	0.0023	0.004–0.017	
PFHpA multiple-adjusted	0.011	0.003	3.13	0.0017	0.004–0.017	0.016
PFHxS sex-adjusted	0.014	0.005	2.77	0.0057	0.004–0.024	
PFHxS multiple-adjusted	0.015	0.005	2.99	0.0028	0.005–0.025	0.012
PFOS sex-adjusted	0.011	0.005	2.35	0.019	0.002–0.020	
PFOS multiple-adjusted	0.011	0.005	2.35	0.019	0.002–0.020	0.0056
PFOA sex-adjusted	0.021	0.006	3.29	0.0010	0.008–0.033	
PFOA multiple-adjusted	0.021	0.006	3.23	0.0012	0.008–0.033	0.0088
PFNA sex-adjusted	0.016	0.006	2.68	0.0074	0.004–0.028	
PFNA multiple-adjusted	0.017	0.006	2.69	0.0072	0.005–0.028	0.0077
PFDA sex-adjusted	0.020	0.007	2.93	0.0034	0.007–0.033	
PFDA multiple-adjusted	0.020	0.007	2.89	0.0038	0.006–0.033	0.0089
PFOSA sex-adjusted	0.011	0.004	2.84	0.0045	0.004–0.020	
PFOSA multiple-adjusted	0.020	0.004	3.06	0.0022	0.005–0.021	0.016
PFUnDA sex-adjusted	0.022	0.007	3.10	0.0020	0.008–0.036	
PFUnDA multiple-adjusted	0.023	0.007	3.26	0.0011	0.009–0.037	0.011

Ln-transformed values for the PFASs are used in the models. The column at the far right shows the change in IMT (mm) that corresponds to a one interquartile range (IQR) change in the PFAS (just given to the multiple adjusted models

PFASs: *PFHpA* perfluoroheptanoic acid, *PFHxS* perfluorohexane sulfonic acid, *PFOS* perfluorooctane sulfonic acid, *PFOA* perfluorooctane sulfonic acid, *PFNA* perfluorononanoic acid, *PFDA* perfluorodecanoic acid, *PFOSA* perfluorooctane sulfonamide, *PFUnDA* perfluoroundecanoic acid

Further adjustments for traditional cardiovascular risk factors changed these relationships only marginally. In these fully adjusted models, baseline PFASs levels were not significantly related to the change in IMT (*p* > 0.05 for all).

As could be seen in Table 4 in detail, a change of one interquartile range (IQR) in the change of PFAS levels (being 0.05 ng/ml for PFHpA, 5.7 for PFHxS, 5.6 of PFOS, 1.3 for PFOA, 0.40 for PFNA, 0.15 for PFDA, 0.09 for PFOSA and 0.19 ng/mlfor PFUnDA) over the 10–year period corresponded to a change in IMT by 0.0056–0.016 mm.

No significant interactions were found between sex and the change in the eight PFASs regarding the change in IMT.

**Fig. 1:**
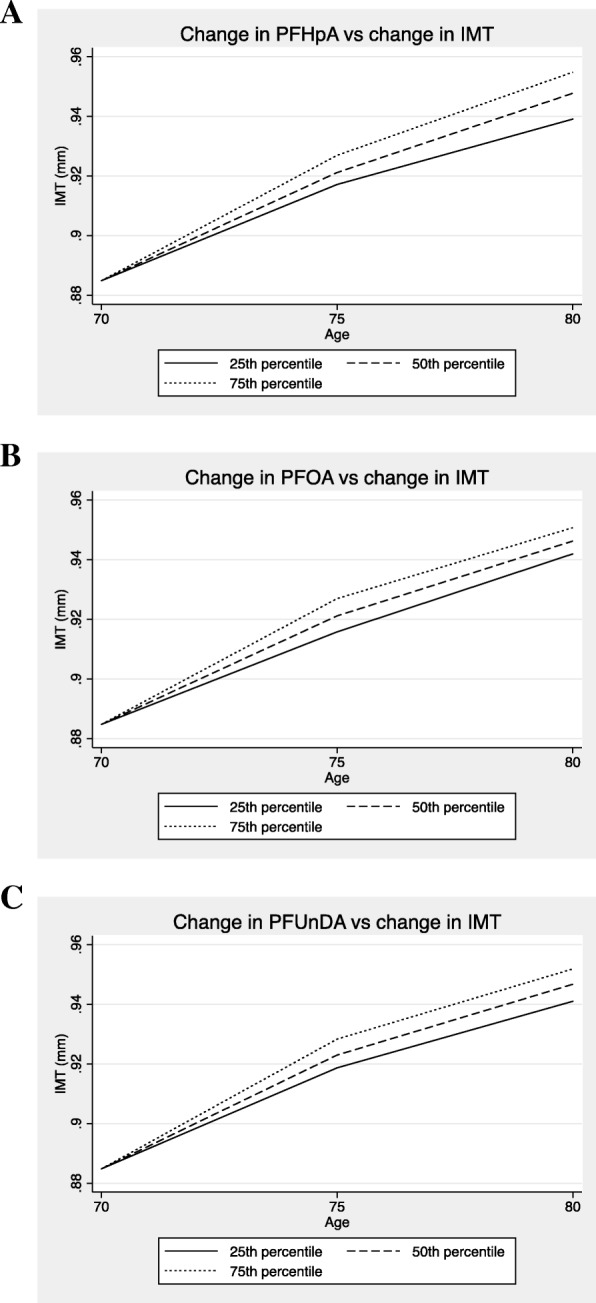
Relationships between the changes in PFHpA (**a**, upper panel), PFOA (**b**, middle panel) and PFUnDA (**c**, lower panel) and the change in carotid intima-media thickness (IMT) from age 70 to age 80 years. The change in the three perfluoroalkyl substances (PFASs) is given for three levels of change, the 25th percentile, the 50th percentile and the 75th percentile. The baseline value (age 70 years) is set the same for all levels of change in PFASs to improve graphic interpretation

The relationships between the changes in plasma levels of the PFASs over the 10 years were related to the change in IMT during the same period. Fig. 1 shows the associations between three different PFASs (PFHpA, PFOA, and PFUnDA) whose chemical structure differs only in the number of carbon-fluorine bonds.

## Discussion

The present longitudinal study showed that the changes in plasma levels of several PFASs were related to the change in IMT during a 10-year follow-up period in a sample of elderly subjects, providing further evidence that PFASs could interfere with the atherosclerotic process. Furthermore, adjustment for the traditional cardiovascular risk factors changed these results only marginally, suggesting that a possible effect of PFASs on IMT is not mediated by the traditional risk factors. In addition, this study adds to previously published studies showing that other environmental contaminants, like the PCBs [20], some phthalates [21], as well as some metals [22], are also linked to atherosclerosis.

In a previous population-based cross-sectional study of Taiwanese adolescents and young adults a positive relationship between PFOS and IMT was found, while the study reported a negative relationship between PFUnDA and IMT [12]. This group also showed that this association was more prominent in individuals with elevated levels of circulating endothelial and platelet microparticles [23]. In the present study, we found that both PFOS and PFUnDA, and some other PFASs, were related to IMT. The reason for this discrepancy between the studies is not known, but might be due to the fact that the studies were conducted in very different age-groups and in countries with different exposures of the PFASs.

Since cross-sectional studies might be subject to reverse causation and confounding, longitudinal studies are preferable. Therefore, the present longitudinal study provides a higher level of evidence that PFASs could interfere with atherosclerosis formation, although only randomized interventions could provide the final evidence in this respect. It is interesting to note that in a cross-sectional analysis of the same study at the baseline investigation at age 70 we did not find any significant relationship between PFASs and IMT [24]. This exemplifies that the longitudinal approach is far superior to the cross-sectional approach, since it also increases the statistical power, as the within-individual variation is usually lower than the between-individual variation.

As a sensitivity analysis, we performed an analysis on the relationships between the changes in PFASs and change in IMT only in the 579 subjects attending all three examinations and the effect estimates, the betas, were almost identical although the confidence intervals were wider due to the small number of observations. Thus, loss of follow-up cannot explain the relationships found in the present study.

The mechanism behind the suggestive effect of PFASs on carotid atherosclerosis is not known. PFASs are known ligands of the nuclear receptors PPAR alpha and gamma [25], and could thereby influence lipids, adipose tissue, and glucose homeostasis. It has previously been shown that pharmaceutical PPAR-gamma ligands, such as thiazolidinediones, could influence IMT [26], so an effect of other PPAR ligands, like PFASs, on IMT is not surprising. It has also been repeatedly shown that PFASs influence cholesterol metabolism [27]. Furthermore, an immune reaction with subsequent inflammation is considered a cornerstone in atherosclerosis development [28]. From that perspective, it is interesting that exposure to PFASs has been associated with a number of perturbations in the immune system in experimental studies [29–31]. Another recently highlighted mechanism of PFASs is that PFASs might influence circulating microparticles, being markers of endothelial dysfunction constituting an early step in atherosclerosis formation [23]. Thus, since many mechanisms could potentially link PFASs exposure to atherosclerosis, we hope that the previous and present findings of a link between PFASs and atherosclerosis in humans will inspire future experimental work to gain further mechanistic insights.

Correlating the changes in the PFASs to each other, we can see that most of those, especially the long-chained PFDA, PFNA and PFUnDA are highly correlated (Table 3). Thus, it is not easy in this setting to establish if the change in a specific PFASs is related to the change in IMT or if it is a class effect. Also in this case, experimental studies are needed.

The fact that correlations generally were seen between the changes in the eight PFASs might explain the fact that a positive association was seen between the change in most of the PFASs studied and the change in IMT despite the fact that some of the PFASs declined over the 10 year period and some increased.

IMT has previously been associated with future myocardial infarction and stroke [11]. In a recent publication, also the predictive power of the change in IMT has been evaluated in a meta-analysis of several studies based on individual data [18] and it was found that baseline IMT was related to cardiovascular events, but not the change in IMT. In that study the follow-up period was shorter (median 4 years) than in the present study (10 years). However, the clinical implications of the found relationships between the change in PFASs and the change in IMT are not obvious.

In the models where both the baseline PFASs levels and the change in PFAS were included, only the change in PFASs were significantly related to the change in IMT. This might indicate that changes in PFASs levels over a fairly short time-frame could be of importance for IMT, but it might also be due to the fact that from a statistical view the within-subject variation (change in PFASs) is by far a more powerful determinant of a change (in IMT) than the between-subject variation (baseline PFASs).

The mean IMT increase over the 10 years was 0.06 mm. Eighty-five percent of this increase occurred during the first 10 years. As given in detail in ref. 17, a general increase in the PFASs were seen during these first 5 years. However, during the second 5-year period, the levels of the PFASs generally went down, which might be an explanation to why the increase in IMT over the last 5 years was very small. During this period, we also noted a decline in IMT in a number of subjects.

The number of carbon-fluorine bonds constituting the different PFASs analogues is a major determinant of the half-life of the PFASs in humans. Overall, experimental studies have reported that both persistence and toxicity tend to increase with increasing numbers of carbon-fluorine bonds. In the present study, eight PFASs consisting of six to eleven carbons (C_6_-C_11_) were evaluated, and six were significantly related to IMT in a similar fashion (see Fig. 1).

We have previously shown that some PFASs levels were related to plaque prevalence. However, we think the change in plaque prevalence over a limited time period is not a good measure of the change in the amount atherosclerosis. For that purpose we need a more graded measure of plaque, like the plaque area, which unfortunately is not available over time in the PIVUS study. Furthermore, in the subjects with 2 carotid plaques (about 1/3 of the population) any progression cannot be evaluated, and this part of the population is probably the most important to study.

The strength of the present study is the repeated measurements of PFASs, traditional cardiovascular risk factors, and IMT at three time points over 10 years in a fairly large sample. One limitation is that the sample consists of elderly Swedes, which limits the generalizability.

## Conclusions

In conclusion, the changes in plasma levels of six out of the eight PFASs evaluated during 10 years were related to the increase in IMT seen during the same period, representing prospective evidence that PFASs might interfere with the atherosclerotic process.

## Abbreviations

AMS: Artery Measurement Software
CCA: Common carotid artery
CV: Cardiovascular
FORMAS: Swedish Research Council for Environment, Agricultural Sciences and Spatial Planning
HDL: High-density lipoprotein
HPLC-MS/MS: Ultra-performance liquid chromatography-tandem mass spectrometry
IMT: Intima-media thickness
LDL: Low-density lipoprotein
LVI: Large volume injection
PFASs: Perfluoroalkyl substances
PFDA: Perfluorodecanoic acid
PFHpA: Perfluoroheptanoic acid
PFHxS: Perfluorohexane sulfonic acid
PFNA: Perfluorononanoic acid
PFOA: Perfluorooctane sulfonic acid
PFOS: Linear isomer of perfluorooctane sulfonic acid
PFOSA: Perfluorooctane sulfonamide
PFUnDA: Perfluoroundecanoic acid
PIVUS: Prospective Investigation of the Vasculature in Uppsala Seniors
SD: Standard deviation
